# METTL8 mRNA Methyltransferase Enhances Cancer Cell Migration via Direct Binding to ARID1A

**DOI:** 10.3390/ijms22115432

**Published:** 2021-05-21

**Authors:** Shin-Ae Lee, Kang-Hoon Lee, Huisu Kim, Je-Yoel Cho

**Affiliations:** Department of Biochemistry, BK21 Plus and Research Institute for Veterinary Science, School of Veterinary Medicine, Seoul National University, Seoul 08826, Korea; siae0812@snu.ac.kr (S.-A.L.); khlee02@snu.ac.kr (K.-H.L.); gmltn1541@snu.ac.kr (H.K.)

**Keywords:** mRNA modification, epigenetic regulation, breast cancer, ARID1A, cancer migration

## Abstract

The association of RNA modification in cancer has recently been highlighted. Methyltransferase like 8 (METTL8) is an enzyme and its role in mRNA m3C modification has barely been studied. In this study, we found that METTL8 expression was significantly up-regulated in canine mammary tumor and investigated its functional roles in the tumor process, including cancer cell proliferation and migration. METTL8 expression was up-regulated in most human breast cancer cell lines tested and decreased by Yin Yang 1 (YY1) transcription factor knockdown, suggesting that YY1 is a regulating transcription factor. The knockdown of METTL8 attenuated tumor cell growth and strongly blocked tumor cell migration. AT-rich interactive domain-containing protein 1A (ARID1A) was identified as a candidate mRNA by METTL8. ARID1A mRNA binds to METTL8 protein. ARID1A mRNA expression was not changed by METTL8 knockdown, but ARID1A protein level was significantly increased. Collectively, our study indicates that METTL8 up-regulated by YY1 in breast cancer plays an important role in cancer cell migration through the mRNA modification of ARID1A, resulting in the attenuation of its translation.

## 1. Introduction

Breast cancer is the most commonly diagnosed cancer in women around the world and is a major cause of cancer-related death. In recent years, the incidence rate has also rapidly been increasing worldwide [1]. Like other cancers, early detection is the most important facet in overcoming breast cancer [2], but at the same time, the development of treatments remains an important task [3]. In recent years, the incidence rate has also rapidly been increasing worldwide [1]. Canines are also prone to cancer development and have a poor prognosis and mortality rate [4]. The main cause of cancer-related death for female canines is the canine mammary tumor, which has epidemiologic, clinical, morphologic, and prognostic features similar to breast cancer. Thus, canine mammary tumor provides a model for breast cancer research [5,6].

Various modifications are known in animal gene transcripts. Up to now, more than 100 RNA modifications have been identified in major types of RNAs including mRNA, tRNA, rRNA, and miRNA; among these, tRNA and rRNA modifications occur most frequently [7]. Moreover, RNA modifications can be grouped according to different types such as, m1A (N1-methyladenosine), m6A(N6-methyladenosine), m5C (5-methylcytosine), mcm5s2U(5-methoxycarbonyl methyl-2-thiouridine), mcm5U(5-methoxycarbonyl methyl uridine), and m7G (7-methylguanosine) [7]. Identification of the reader and eraser proteins for all these different modifications is very important to better understand the function of each modification [7]. The m6A mRNA modification, one of the most well-known mRNA modifications, is known to be caused by METTL3 and METTL14 and plays an important role in human carcinogenesis [8]. In breast cancer, overexpressed METTL3 has been shown to increase HBXIP mRNA methylation, thereby promoting breast cancer cell proliferation [9].

Methyltransferase like 8 (METTL8) encodes methyltransferase which causes an m3C modification on the target mRNA [10]. It is known that METTL8 regulates differentiation in mouse embryonic stem cells [11]. Gu et al. identified METTL8 during the stem cell development stage and demonstrated that METT8 expression was regulated by STAT3 and might methylate a list of developmental genes in mouse embryonic stem cells. Of note, a few recent studies have reported that mRNA modification does not play a role as cancer driver, but is known to be associated with cancer survival, growth, and differentiation [12]. Recently, SUMOylation of METTL8 has been revealed to induce its nuclear localization, the m3C modification of mRNA, and the formation of an R-loop, which leads to a transcriptional pause resulting in tumorigenesis [13]. Moreover, the frameshift mutations of METTL8 play an important role in colon cancer tumorigenesis [14]. However, more research on its functions and regulation is mandatory because major regulators and target RNAs might be varied in different cells and conditions. In addition, the cancer cell migration and proliferation functions of METTL8 that increase the mortality rate and promote metastasis in breast cancer have not been demonstrated yet [14,15,16]. 

In this study, we investigated the functions of METTL8 in breast cancer. Our results suggest that the YY1-METTL8-ARID1A axis has an important role in breast cancer migration as well as in proliferation. 

## 2. Results

### 2.1. METTL8 Expression Was Up-Regulated in Breast Cancer and Canine Mammary Tumor

**Figure 1:**
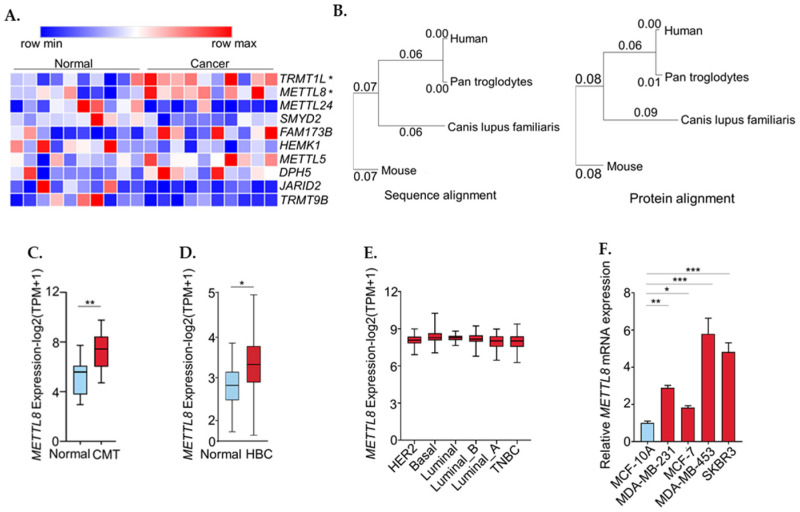
METTL8 expression in public dataset. (**A**) A list of 10 genes with significantly up-regulated expression in canine mammary tumor among 400 methyltransferases. Expression level of genes are represented as log2 (TPM+1). (**B**) METTL8 mRNA sequence and protein similarity between human and canine. (**C**,**D**) Boxplot showing relative expression of METTL8 in canine mammary tumor (**C**) and in breast cancer (**D**). (**E**) Boxplot showing relative expression of METTL8 in breast cancer subtype. (**F**) METTL8 transcript levels. Total RNA extracted from MCF-10A, MDA-MB-231, MDA-MB-453 and SKBR3 cells lines and subjected to RT-qPCR analysis for METTL8. * indicates *p* < 0.05, ** *p* < 0.01, and *** *p* < 0.001, CMT: canine mammary tumor, HBC: human breast cancer.

We analyzed the expression levels of the genes that have been identified as RNA modification-associated functions from the transcriptome data of canine mammary tumor and adjacent normal tissue [17]. A list of 229 methyltransferases in a dog was obtained from the Ensemble database (https://www.ensembl.org; accessed on 11 April 2019) and 225 expressions were confirmed in canine mammary tumor transcriptome (Supplementary Table S1). A list of 10 genes was significantly up-regulated in canine mammary tumors among 225 methyltransferases (Supplementary Table S2). The heatmap presented the expression pattern of the top 10 genes (Figure 1A). Out of the 10 genes related to methyltransferases that were significantly up-regulated in canine mammary tumors, TRMT1L and METTL8 are RNA methyltransferases. Since TRMT1L, which encodes tRNA methyltransferase, has been well-known as a tRNA modifier, we targeted METTL8 in this study.

The higher mRNA and protein sequences similarity of METTL8 between human and dog compared to between human and mouse allows us to assume that the function of METTL8 is more conserved between dog and human (Figure 1B) [11]. METTL8 expression in canine mammary tumor and breast cancer was visualized in boxplots using our previous publication and public database (http://gepia.cancer-pku.cn; accessed on 20 May 2020) (Figure 1C,D). When compared the RNA expression levels in six different breast cancer subtypes, (Her2+, basal, Luminal, Luminal_A, Luminal_B, and triple-negative), METTL8 is evenly expressed in all of them (Figure 1E).

However, METTL8 expression was varied in five different breast-associated cell lines, MCF10A (normal breast cell), MDA-MB-231(triple-negative), MCF7 (ER+), MDA-MB-453 (HER2+), and SKBR3 (HER2+). Overall, METTL8 expression was higher in all the cancer cell lines than in the normal cell line. MDA-MB-435 showed the highest expression followed by SKBR3; both are originated from the HER2+ subtype (Figure 1F). Collectively, the up-regulation of METTL8 in breast cancer is not limited to specific cell types and also occurs in various cancers, including canine mammary tumors in dogs.

### 2.2. YY1 Is an Important Transcription Factor of METTL8 in Breast Cancer

**Figure 2:**
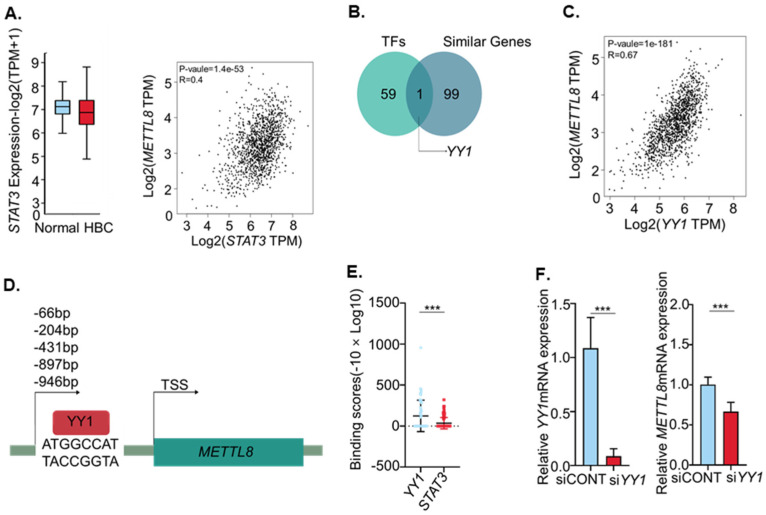
(**A**) Boxplot showing relative expression of STAT3 in breast cancer. The correlation between STAT3 and METTL8 expression in breast cancer was analyzed in gepia2 (http://gepia2.cancer-pku.cn; accessed on 20 May 2020). (**B**) Venn diagram showing the intersection of genes whose expression is genes similar in expression to expression of METTL8 in breast cancer from TCGA and/or GTEx expression data accessed via gepia2 (http://gepia2.cancer-pku.cn; accessed on 20 May 2020) with the genes identified as METTL8 putative transcription factors by PROMO (http://alggen.lsi.upc.es; accessed on 20 May 2020). (**C**) The correlation between YY1 and METTL8 expression in breast cancer was analyzed in gepia2 (http://gepia2.cancer-pku.cn; accessed on 20 May 2020). (**D**) The binding site of transcription factor YY1 is estimated by PROMO (http://alggen.lsi.upc.es; accessed on 20 May 2020). (**E**) Peak-call data of the same antigens were collected from public datasets, and MACS2 scores (−10 × Log10 (MACS2 Q-value)) are displayed binding scores for METTL8. (**F**) YY1 and METTL8 mRNA levels were analyzed by RT-qPCR 48 h after YY1 knocked-down in MDA-MB-231 cells as compared with the control. *** indicates *p* < 0.001, HBC: human breast cancer.

Since the regulation of gene expression by transcription factors (TFs) has very dynamic mechanisms in which various TFs are orchestrated, we endeavored to determine the major regulator of METTL8 expression in breast cancer. STAT3 suggested as a METTL8 transcription factor in the previous study [11], is shown to have a lower correlation than YY1 in breast cancer. Additionally, STAT3 expression was slightly lower in breast cancer patients (Figure 2A). Therefore, we extracted the genes of which the expressions are positively correlated with METTL8 in breast cancer from TCGA and/or GTEx expression data using gepia2 (http://gepia2.cancer-pku.cn; accessed on 20 May 2020). Simultaneously, we searched for transcription factor binding sites on the METTL8 promoter region using PROMO (http://alggen.lsi.upc.es; accessed on 20 May 2020). The Venn diagram showed a total of 100 correlated expression genes and 60 TFs with possible binding to the promoter region, and the integration of these two lists produced only one transcription factor, YY1 (Figure 2B). In addition, when we compared the correlation of the gene expression patterns between METTL8 and putative transcription factors, YY1 showed a better correlation coefficient in breast cancer compared to other putative transcription factors. Unexpectedly we could not find a STAT3 binding site on the METTL8 promoter region (Supplementary Table S3 and Figure 2C). Putative YY1 binding sites were identified at 946 bp, 897 bp, 431 bp, 204 bp and 66 bp (starting position) upstream of the transcription start sites (TSS) of METTL8 (Figure 2D). Of note, the YY1 binding stronger than STAT3 on the chromosomal region of the METTL8 gene was confirmed from the ChIP-Atlas dataset (http://chip-atlas.org; accessed on 20 May 2020). YY1 (ave. 125.7) binding score is approximately four times higher than STAT3 (ave. 34.4) on the METTL8 gene (−10 × Log10 (MACS2 Q-value)). This result suggests that YY1 contributes to the METTL8 expression regulation. To validate the regulation of YY1 on METTL8 transcription in breast cancer, we tested the expression level of METTL8 mRNA in silenced YY1 conditions. This may support our result showing that, instead of STAT3 (Gu et al., 2018), YY1 is a more important transcription factor regulating METTL8 expression in breast cancer. Treatment of siYY1 significantly eliminated YY1 mRNA at the 48 h time point (*p* < 0.05). At the same time, METTL8 mRNA expression was also significantly decreased in siYY1 treated cells (Figure 2F). 

### 2.3. METTL8 Knockdown Decreased Proliferation and Migration of Breast Cancer Cell Line

**Figure 3:**
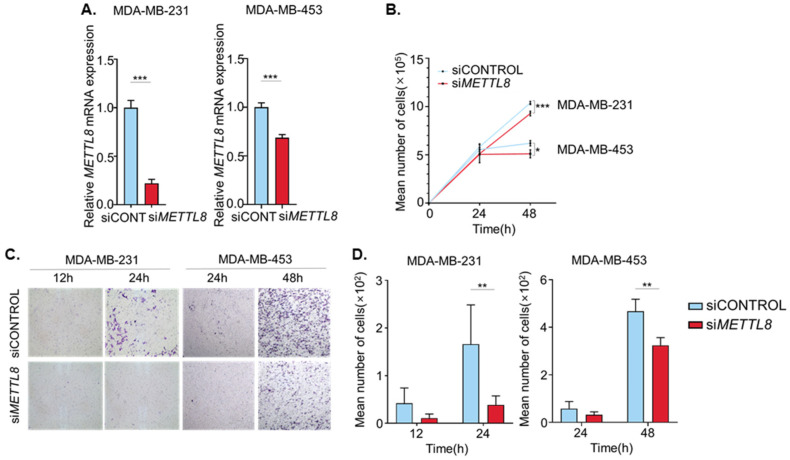
(**A**) Knockdown efficiencies of METTL8 in both MDA-MB-231 and MDA-MB-453 cell lines were verified by RT-qPCR. (**B**) Cell proliferation of METTL8 knockdown cells and negative control was assessed by cell counting. (**C**,**D**) Cell migration of METTL8 knockdown cells and negative control cells were measured by transwell assay. * indicates *p* < 0.05, ** *p* < 0.01 and *** *p* < 0.001.

To investigate the function of METTL8 in breast cancer cell lines, METTL8 expression was knock-downed in breast cancer cell lines using siRNAs, and the knockdown of METTL8 transcripts was confirmed by quantitative RT-PCR. METTL8 transcript expression was decreased significantly in both MDA-MB-231 and MDA-MB-453. (Figure 3A). The effect of METTL8 knockdown on cell proliferation, as measured by the mean number of cells, showed slightly delayed growth compared with the controls in both cell lines at 48 h after transfection (Figure 3B). Importantly, cancer cell migration was also significantly impeded by METTL8 knockdown in both breast cancer cell lines (Figure 3C). Only 20% of MDA-MB-231 cells transfected with siMETTL8 were migrated through the filter when compared with siControl at a 24 h time point. Similarly, but slowly in MDA-MB-453, knockdown of METTL8 decreased cancer cell migration up to 30% at the 48 h time point (Figure 3D). Taken together, these results indicate that knockdown of METTL8 reduces breast cancer cell proliferation and migration in vitro. 

### 2.4. METTL8 Interacts with ARID1A mRNA

**Figure 4:**
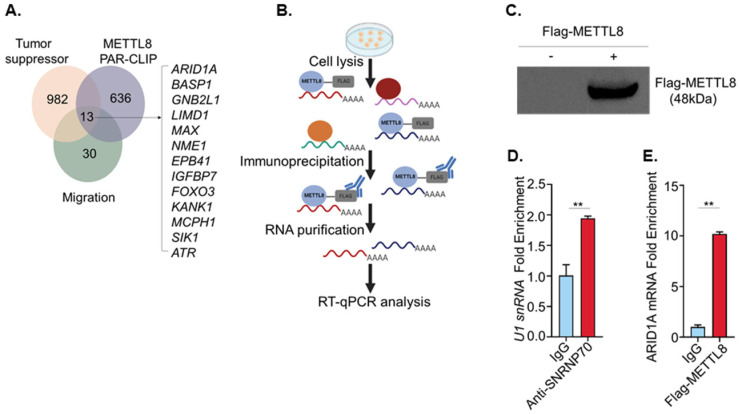
(**A**) Venn diagram showing mRNAs that bind to METTL8 via a putative target gene list of METTL8(11), a tumor suppressor genes list (18), and a list of cancer migration-related genes. (**B**) Schematic summary of the RIP protocol followed by RT-qPCR were created in BioRender.com. (**C**) Western blot shows the expression of Flag-tagged METTL8 protein in the RIP lysate. (**D**) RT-qPCR detection of positive control U1 snRNA in RNAs enriched by anti-snRNP70 RIP. ** indicates *p* < 0.01. (**E**) ARID1A mRNA was highly enriched in the RNAs enriched by anti-Flag METTL8 RIP by RT-qPCR. MDA-MB-231 cells were subjected to RIP assay after transfection with Flag-tagged METTL8 cDNA ORF Clone. ** indicates *p* < 0.01.

To identify putative METTL8 target transcripts associated with cancer cell proliferation and migration phenotypes, we surveyed the literature fulfilling three criteria: (i) tumor suppressor genes [18], (ii) METTL8 binding transcripts [11] and (iii) genes associated with cancer cell migration. Gu et al. initially identified METTL8 and documented its mRNA methyl-transferase function during the mouse embryonic development process. A total of 649 transcripts were captured by the METTL8 protein and were suggested as putative target genes [11]. The Venn diagram in Figure 4A presents a total of 13 genes (ARID1A [19], BASP1 [20], GNB2L [21], MAX [22], LIMD1 [23], NME1 [24], EPB41 [25], IGFBP7 [26], FOXO3 [27], KANK1 [28], MCPH1 [29], SIK1 [30], and ATR [31]) that satisfy the three criteria. We then finally chose ARID1A based on the discrepancy between the expression level of mRNA and protein in breast cancer and canine mammary tumor databases because the function of METTL8 is mRNA methylation, which is known to result in restrained protein expression. Although ARID1A has been known as a tumor suppressor, mRNA expression of ARID1A is still higher in breast cancer than in normal control. However, protein level was not significantly changed in breast cancer (the human protein atlas; https://v15.proteinatlas.org/ENSG00000117713-ARID1A/cancer; accessed on 20 May 2020). 

To investigate whether METTL8 interacts with ARID1A mRNA in breast cancer cells, we performed an RNA immunoprecipitation assay (RIP). As shown in Figure 4B, METTL8-ARID1A mRNA complexes were isolated by immunoprecipitation using a Flag antibody. The mRNAs bound to METTL8 protein were then analyzed by RT-qPCR after confirming the transfected METTL8-Flag expression from the cell lysate (Figure 4C). To ensure RIP experiment assurance, we also performed the pulldown of the known SNRNP70-bound U1 snRNA as a control using an anti-SNRNP70 antibody (Figure 4D). ARID1A mRNA was nine times more enriched in anti-flag METTL8 than IgG control, indicating the direct binding of METTL8 to ARID1A mRNA (Figure 4E). Indeed, the direct interaction between METTL8 and ARID1A mRNA allows us to deduce that ARID1A mRNA can be methylated by METTL8 protein.

### 2.5. ARID1A mRNA Is a Candidate Transcript Associated with the Cell Migration Phenotype Regulated by METTL8

**Figure 5:**
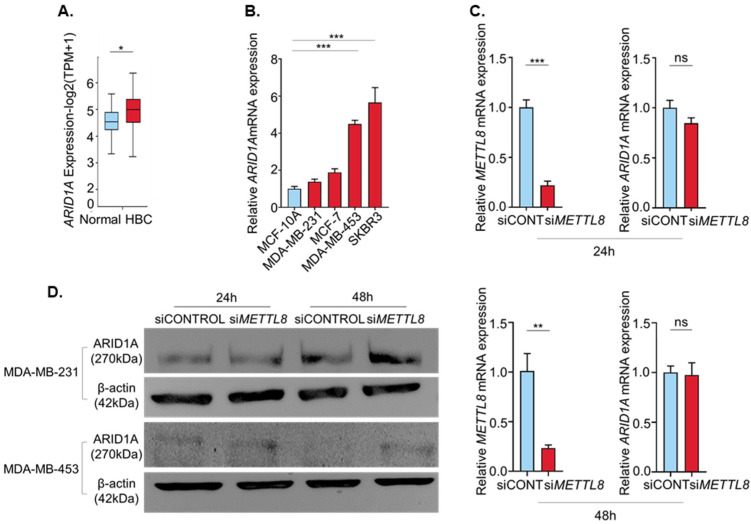
(**A**) Boxplot showing relative expression of ARID1A in breast cancer. (**B**) Total RNAs were extracted from MCF-10A, MDA-MB-231, MDA-MB-453, and SKBR3 cell lines and subjected to RT-qPCR analysis for ARID1A. (**C**) ARID1A mRNA levels were not changed by METTL8 knockdown. METTL8 and ARID1A mRNA levels were analyzed by RT-qPCR at 24 h and 48 h after the METTL8 knockdown in MDA-MB-231 cells. (**D**) ARID1A protein expression levels were increased after METTL8 knockdown. Western blots were subjected to detect ARID1A protein expression 24 h and 48 h after METTL8 siRNA transfection in MDA-MB-231 cells and MDA-MB-453 cells. * indicates *p* < 0.05, ** *p* < 0.01, and *** *p* < 0.001, HBC: human breast cancer.

ARID1A expression was higher in breast cancer than in the normal (Figure 5A). We thus investigated the regulation of ARID1A protein expression via METTL8 induced mRNA modifications. Interestingly, the ARID1A gene expressions in four breast cancer cell lines were higher than the normal cell line. Additionally, similar to Figure 1F, two breast cancer cell lines, MDA-MB-453 and SKBR3 showed a significantly high ARID1A expression than MCF10A (Figure 5B). We first showed that METTL8 does not affect ARID1A mRNA expression. As expected, the ARID1A mRNA expression was not significantly changed by the silencing of METTL8 by siMETTL8 at both 24 h and 48 h after transfection (Figure 5C). However, the ARID1A protein level was dramatically increased in siMETTL8 treated cells. The ARID1A protein was slightly but consistently increased in siMETTL8 treated cells compared with siCONTROL at 24 h after transfection, and highly increased at the 48-h time point in both MDA-MB-231 and MDA-MB-453 (Figure 5D). Taken together our results suggest that METTL8 modulates ARID1A protein expression via mRNA modification.

## 3. Discussion

Recently, clinical and molecular similarities between human breast cancer and canine mammary tumors have been largely identified from many studies [32]. Not only the clinical similarities including the spontaneous tumor incidence, onset age, course of the disease, and clinical-stage but also a number of molecular markers for human breast cancer such as BRCA gene mutations, EGF receptor (EGFR), Ki-67, human epidermal growth factor receptor (HER2)/neu, p53, p63, and matrix metalloproteinases were also demonstrated to serve an essential role in canine mammary tumors. We conducted our study based on the similarity between human breast cancers and canine mammary tumors and elucidated the role of METTL8 in human breast cancer.

With the increasing importance of epigenetic regulations in recent cancer research, messenger RNA modification related to cancer survival, proliferation, growth, and differentiation has been emphasized [12]. Chromosomal DNA methylation is the most well-known epigenetic modification, but little has been done with RNA methylation, especially with mRNA methylation. Therefore, the functional study of METTL8, one of the mRNA modifiers will be very important for a better understanding of breast cancer. In this study, we confirmed that METTL8 was increasing in breast cancer of human and dog, and revealed its functional roles. 

We first showed that the expression of METTL8 is regulated by the YY1 transcription factor. YY1 is highly expressed in breast cancer and cooperates with activator protein to stimulate the expression of ERBB2 (Her2/neu), a proto-oncogene overexpressed in approximately 30% of breast cancers [33]. In addition, YY1 regulates the expression of many cancer-related genes such as MYC (alias c-myc) and c-Fos [34]. Although the contradictory functions of YY1 have been reported as a transcription activator and repressor, in our study, YY1 seems to work as the transcription activator of METTL8. 

Although it has been suggested that STAT3 is the regulator of METTL8 gene expression in mouse embryonic development, it is an important result of showing that METTL8 expression in humans, especially in breast cancer conditions, is caused by not STAT3, but the YY1 transcription factor [11]. Although STAT3 binding signals are still observed in the METTL8 gene by ChIP-Atlas, the STAT3 binding site was not found within 1000 bp of the transcription start site of the METTL8 gene. Simply, it might be explained that STAT3 binding sites exist outside of the range surveyed or the METTL8 promoter sequences are different between mouse and human. Otherwise, these results can appear to differential TF binding among cell lines not only by differences in chromatin accessibility, but also by cell type-specific primary sequence preferences [35]. Indeed, the binding of YY1 protein on the METTL8 gene was confirmed by public ChIp-seq datasets.

We also demonstrated that METTL8 can affect the proliferation and migration of cancer cells. Interestingly, METTL8 knockdown in both MDA-MB-231 and MDA-MB-453 cells significantly reduced cell proliferation and migration capabilities. Furthermore, we showed how METTL8 affects the phenotype of breast cancer cells by identifying ARID1A as a target gene for METTL8. We showed that the METTL8 protein binds directly to ARID1A mRNA, and the inhibition of METTL8 expression significantly increases ARID1A protein levels without changing the amount of mRNA in ARID1A. Here, the post-transcriptional regulation of METTL8 on the ARID1A expression might be the answer for how ARID1A expression can be high in the cells of which METTL8 expression is high (Figure 1F and Figure 5B). Since METTL8 works on the ARID1A transcripts at the post-transcriptional level to suppress ARID1A translation, although ARID1A transcripts were high level in the HER2 cell lines that highly express METTL8, as shown in our Western blot data, ARID1A protein can be repressed. 

The involvement of ARID1A in cancer progression is known to regulate the PI3K/AKT pathway associated with cancer cell growth, proliferation, and migration as well as a tumor suppressor role [36,37]. ARID1A (AT-rich interacting domain-containing protein 1A gene) is a subunit of SWI/SNF (the Switch/Sucrose Non As a subunit of -Fermentable), a chromatin remodeling complex, and it recruits other components to the SWI/SNF complex for chromatin remodeling [38,39]. Mutations that disable ARID1A occur in several cancers, including gastric cancer, hepatocyte cancer, breast cancer, and pancreatic cancer, and ARID1A mutations interfere with tumor suppression and cause carcinogenesis through other mechanisms [37,40]. In endometrial carcinomas, the PI3K-AKT pathway is activated by an ARID1A mutation [41]. The activation of the PI3K-AKT pathway increases cancer cell proliferation and migration [36,37]. 

In conclusion, this study presented a new role of METTL8 in breast cancer and identified possible mechanisms. Although it still has some limitations such as not directly showing the mRNA methylation of ARID1A and modulation of PI3K-AKT pathway via METTL8, this study can be an important clue to better understand the epigenetic regulation of breast cancer.

## 4. Materials and Methods

### 4.1. Cell Culture and Transfection

MDA-MB-231(triple-negative), MCF7 (ER+), MDA-MB-453 (HER2+), and SKBR3 (HER2+) were obtained from the Korean cell line bank. MDA-MB-231(triple-negative), MCF7 (ER+), MDA-MB-453 (HER2+) were cultured in DMEM/high glucose medium (Hyclone, Logan, UT, USA) supplemented with 100% US origin fetal bovine serum (FBS) (Hyclone, Logan, UT, USA), and 1% antibiotic–antimycotic (100×) (Gibco, Gaithersburg, MD, USA). SKBR3 (HER2+) was cultured in RPMI 1640 medium (Hyclone, Logan, UT, USA) supplemented with 100% US origin fetal bovine serum (FBS) (Hyclone, Logan, UT, USA), and 1% antibiotic–antimycotic (100×) (Gibco, Gaithersburg, MD, USA). Breast epithelial cell line MCF-10A cells were cultured in MEBM medium (Lonza, Allendale, NJ, USA), supplemented with BPE (13 mg/mL), hydrocortisone (0.5 mg/mL), hEGF (10 µg/mL), insulin (5 mg/mL), 100% US origin fetal bovine serum (FBS) (Hyclone, Logan, UT, USA) and 1% antibiotic–antimycotic (100×) (Gibco, Gaithersburg, MD, USA). All cell lines were cultured at 37 °C in a 5% CO_2_ atmosphere.

A total of 50 pmol of each siRNA (Bioneer, Daejeon, South Korea), SDO-1001 for METTL8, YY1, and SN-1012 for the negative control, were transfected with 5 µL of Lipofectamine RNAiMAX transfection reagent (Thermo Fisher Scientific, Waltham, MA, USA) according to the manufacturer’s instructions. Cells were harvested at 24 and 48 h after incubation for further analysis.

Flag-tagged METTL8 vector (Sinobiological, Beijing, China), was transfected with Lipofectamine 3000 transfection reagent and P3000 (Invitrogen, Shanghai, China) according to the manufacturer’s instructions. Cells were harvested at 24 h after incubation for further analysis.

### 4.2. RNA Immunoprecipitation(RIP)

RIP assay was conducted using the Magna RIP RNA-Binding Protein Immunoprecipitation Kit (Millipore, Billerica, MA, USA) according to the manufacturer’s protocol. MDA-MB-231 cells were transfected with a Flag-tagged METTL8 vector (Sinobiological, Beijing, China). A total of 2 × 10^7^ cells was used per RIP. FLAG antibody (Cell Signaling, Danvers, MA, USA) 10 µg or positive control anti-SNRNP70 (Millipore, Billerica, MA, USA) 5 µg and magnetic beads were added to the cell lysate and incubated overnight at 4 °C under gentle rotation. Afterward, the immunoprecipitated RNA was isolated, purified, and subjected to RT-qPCR analysis.

### 4.3. RNA Extraction and Quantitative RT-PCR

RNA extraction and quantitative RT-qPCR were done as previously reported [17]. Briefly, total RNAs were extracted from cells with Trizol (Life Technologies, Carlsbad, CA, USA). Total RNA (2 µg) was reverse transcribed into cDNA using a CellScript All-in-One 5× First Strand cDNA Synthesis Master Mix (CDS-100, CellSafe, Gyeonggi-do, South Korea) according to the manufacturer′s instruction. For RIP assay, purified RNA was reverse transcribed using the Omniscript RT kit (Qiagen, Valencia, CA, USA) with 1 µL of Random primer (nonadeoxyribonucleotide mixture; pd (N)9) (Takara, Kyoto, Japan) per reaction. RT-qPCR was performed using the Real-Time qPCR detection system (CFX Manager™ Software, Bio-Rad) with SYBR Premix Ex Taq™ (Tli RNaseH Plus) (Takara, Kyoto, Japan). RT-qPCR was performed for 35 cycles of 95 °C for 20 s, 62 °C for 20 s, and 72 °C for 20 s. The primers used for real-time RT-qPCR were listed in Table 1. The samples were run in triplicate and the expression of each target gene mRNA level was analyzed using the 2-ΔΔCT method and normalized to human CCSER2 expression. 

### 4.4. Protein Extraction and Western Blot

Protein extraction and Western blot were performed as previously reported in our group [42]. Briefly, total proteins were extracted from cells in RIPA lysis buffer (Thermo Fisher Scientific, Waltham, MA, USA) and subjected to SDS-PAGE. For METTL8 and β-Actin analysis, the proteins were separated by 10% SDS-PAGE for 120 min, while for ARID1A the proteins were separated by 6% SDS-PAGE for 120 min. After transferring to nitrocellulose membrane (NC), the proteins were probed with the primary antibodies of anti-β-Actin (1:2000) (Sigma-Aldrich, Zwijndrecht, The Netherlands), and rabbit anti-ARID1A (1:1000) (Cell Signaling, Danvers, MA, USA). After secondary antibody incubation, the proteins were detected by chemiluminescence using Pierce™ ECL plus Western blotting substrate (Thermo Fisher Scientific, Waltham, MA, USA).

### 4.5. Cell Proliferation Assay

Cell proliferation was determined by cell counting using a hemocytometer and Trypan blue solution, 0.4% (Gibco, Gaithersburg, MD, USA). For cell counting, the cells from 6 well plates (Corning, NY, USA) were harvested with 0.25% trypsin-EDTA (Gibco, Gaithersburg, MD, USA) and centrifuged at 1000 rpm for 3 min (HANIL SCIENCE MEDICAL). The cell pellet was resuspended in 1 mL PBS/phosphate buffered saline solution (HyClone, Logan, UT, USA) and stained with trypan blue. The cell number (cells/mL) was counted under a microscope (Macrotech, Gyeonggi-do, Korea) from 24 h to 48 h after transfection.

### 4.6. Cell Migration Assay

Cell migration assay was performed as previously reported in our group [43]. Briefly, the assay was performed using Corning transwell polycarbonate membrane cell culture inserts (Costar, Cambridge, MA, USA) in 24-well dishes. For MDA-MB-231, approximately 5 × 10^4^ cells in 200 µL of DMEM/high glucose medium (Hyclone, Logan, UT, USA) supplemented with 100% US origin fetal bovine serum (FBS) (Hyclone, Logan, UT, USA) were placed in the chamber, and 500 µL of the same medium was added in the chamber. In MDA-MB-453, approximately 1.2 × 10^5^ cells in 100 µL of DMEM/high glucose medium (Hyclone, Logan, UT, USA) supplemented with 100% US origin fetal bovine serum (FBS) (Hyclone, Logan, UT, USA) were placed in the chamber, and 500 µL of the same medium was added in the chamber. The plates were incubated at 37 °C in 5% CO_2_. After washing, cells were fixed and stained with crystal violet. Cells were counted under a microscope using the ToupView program.

### 4.7. Public Data Acquisition

The list of Methylotransferase was obtained from Ensembl database (http://ensembl.org; accessed on 11 April 2019). Both coding sequence and amino acid sequence of METTL8 gene in four different species were downloaded from NCBI database (https://www.ncbi.nlm.nih.gov/; accessed on 20 May 2020) and sequence alignment and phylogenic analysis was performed using distance tree results of BLAST. Boxplot data confirming the expression of METTL8 for each breast cancer subtype was analyzed by gent2 (http://gent2.appex.kr/gent2/; accessed on 20 May 2020). Data showing the sequence similarity of METTL8 between canine and human were acquired from NCBI (https://www.ncbi.nlm.nih.gov/; accessed on 20 May 2020). A boxplot showing the expression difference of METTL8 between normal and breast cancer (BRCA), STAT3-METTL8 and YY1-METTL8 pair-wise gene correlation analysis (spearman), as well as a list of genes with expressions similar to METTL8 in breast cancer was acquired in gepia2 (http://gepia2.cancer-pku.cn; accessed on 20 May 2020). A list of putative transcription factor binding sites of METTL8 was analyzed in PROMO (http://alggen.lsi.upc.es; accessed on 20 May 2020). YY1 and STAT3 of binding scores for METTL8 were acquired in ChIP-Atlas (http://chip-atlas.org; accessed on 20 May 2020). Graphical Abstract was created in BioRender.com.

### 4.8. Gene Expression Database Analysis

Our previous RNA-seq dataset of dog mammary gland tumors and adjacent normal tissues [17] was reanalyzed and used for this study (https://doi.org/10.3390/cancers10090317; accessed on 20 May 2020). From these data, a heat map was drawn to confirm the expression of the gene that causes RNA methylation in normal and mammary gland tumors.

## Figures and Tables

**Table 1:** Primers for RT-qPCR analysis.

Gene	Direction	Sequence
METTL8	Forward	5′-CGAGGAGATGGTACCAGAGCATA-3′
	Reverse	5′-AGCGGCGATCAACCAGATTT-3′
YY1	Forward	5′-AAAACATCTGCACACCCACG-3′
	Reverse	5′-GTCTCCGGTATGGATTCGCA-3′
ARID1A	Forward	5′-CTTCAACCTCAGTCAGCTCCCA-3′
	Reverse	5′-GGTCACCCACCTCATACTCCTTT-3′
CCSER2 (control)	Forward	5′-GACAGGAGCATTACCACCTCAG-3′
	Reverse	5′-CTTCTGAGCCTGGAAAAAGGG-3′
18S (control)	Forward	5′-AACCCGTTGAACCCCATT-3′
	Reverse	5′-CCATCCAATCGGTAGTAGCG-3′

## Data Availability

Not applicable.

## Supplementary Materials

The following are available online at https://www.mdpi.com/article/10.3390/ijms22115432/s1, Table S1: Gene expression for 225 methyltransferases in the RNA-seq data of canine mammary tumor tissue and adjacent normal tissues, Table S2: Ten methyltransferases genes significantly up-regulated in canine mammary tumor, Table S3: Putative transcription factor binding sites on the METTL8 promoter region (−1 kb) provided by PROMO.
